# Levels of Contamination by Perfluoroalkyl Substances in Honey from Selected European Countries

**DOI:** 10.1007/s00128-016-1840-5

**Published:** 2016-05-27

**Authors:** Magdalena Surma, Henryk Zieliński, Mariusz Piskuła

**Affiliations:** Malopolska Centre of Food Monitoring, Faculty of Food Technology, University of Agriculture in Krakow, 122, Balicka str., 30-149 Krakow, Poland; Department of Chemistry and Biodynamics of Food, Division of Food Science, Institute of Animal Reproduction and Food Research of the Polish Academy of Sciences, 10, Tuwima str., 10-748 Olsztyn, Poland

**Keywords:** PFASs, PFCAs, PFSAs, *d*-SPE, Honey, micro-HPLC–MS/MS

## Abstract

Perfluoroalkyl substances (PFASs) are man-made chemicals manufactured for numerous applications. The aim of this study was to assess the levels of 10 PFASs in selected types of honey samples from selected eastern, northern and southern European countries. A total of 26 samples of honey were analyzed. PFCAs (perfluoroalkyl carboxylic acids) were detected in almost all (92 %) analyzed samples in the range of 0.124–0.798 ng g^−1^ ww (wet weight). The average concentrations of particular PFCAs (ng g^−1^ ww) in honey samples increased in the following order: perfluorononanoic acid (0.164) < perfluorooctanoic acid (0.189) < perfluoroheptanoic acid (0.271) < perfluorodecanoic acid (0.278). Amongst perfluoroalkane sulfonates, only perfluorohexane sulfonate (PFHxS) was identified in four of 26 analyzed samples, and its concentrations ranged from 0.080 to 0.191 ng g^−1^ ww. Italian eucalyptus honey contained the highest total content of PFASs (0.878 ng g^−1^ ww). Samples originating from an industrial region of Poland showed 20 % higher concentrations of PFCAs compared to those from non-industrial regions.

Perfluoroalkyl substances (PFASs) are a group of organofluorine compounds, i.e., aliphatic hydrocarbons with all or almost all hydrogen atoms replaced with fluorine. PFASs can be distinguished into two main groups: perfluoroalkyl carboxylic acids (PFCAs) and perfluoroalkane sulfonates (PFSAs) (Buck et al. 2011). Due to the strength of the C-F bond, they are highly chemically stable and highly resistant to biological degradation. Therefore, these compounds tend to persist in the environment and have been proposed as a new class of persistent organic pollutants (Stahl et al. 2011). Human exposure to PFASs can be due to a variety of environmental and product-related sources. The greatest portion of chronic exposure to PFASs has been suggested to be the result of intake of contaminated foods, including drinking water (Del Gobbo et al. 2008; Domingo 2012; Ericson et al. 2008; Gellrich et al. 2013; Kärrman et al. 2007; Zhao et al. 2012).

Honey is a natural food, composed mainly of a complex mixture of carbohydrates and other minor substances such as organic acids, amino acids, proteins, minerals, vitamins and lipids (Finola et al. 2007). Honey is produced by honey bees from the nectar of blossoms or from the secretions of living parts of plants. It is also an inexpensive product available for novel therapies against bacterial infections (Huttunen et al. 2012). The clinical use of honey has enormous potential, especially in the fight against antibiotic-resistant strains (Huttunen et al. 2012; Kwakman et al. 2008; Mercan et al. 2007). Due to the world-wide consumption of honey, especially among children, there is a demand for honey that is free from contaminants such as persistent organic pollutants, including the compounds of interest in this study. The specific composition of any batch of honey, including contaminants, depends on the crops surrounding the beehive (Aliferis et al. 2010; Kujawski and Namieśnik 2008). The occurrence of PFASs in honey results from bees collecting nectar from contaminated flowers (Celli and Maccagnani 2003), which in turn can be contaminated by soil, air and water. Therefore, honey may serve also as an indicator of environmental pollution by PFASs.

Currently, there is no legislation concerning PFASs in food or feed within the EU (EFSA 2008). The limited scope of research and preliminary findings of EFSA indicate the need to identify PFASs in raw material as well as in food of plant origin in order to reveal the present scale of their occurrence and the associated risk to human health (Surma and Zielinski 2015). To estimate the extent of their presence in food, in March 2010, Commission Recommendation 2010/161/EU invited the Member States to monitor the presence of perfluorooctane sulfonates (PFOSs) and perfluorooctanoic acids (PFOAs) (compounds similar to PFASs but with different chain lengths) and their precursors (EU Recommendation No. 161, 2010).

The aim of this study was to assess levels of PFAS-contamination in honey samples from selected eastern, northern and southern European countries. Amongst PFASs, selected perfluoroalkyl carboxylic acids (PFCAs), such as perfluorobutanoic acid (PFBA), perfluoropentanoic acid (PFPeA), perfluorohexanoic acid (PFHxA), perfluoroheptanoic amid (PFHpA), perfluorooctanoic acid (PFOA), perfluorononanoic acid (PFNA) and perfluorodecanoic acid (PFDA), as well as perfluoroalkane sulfonates (PFSAs) such as perfluorobutane sulfonate (PFBS), perfluorohexane sulfonate (PFHxS) and perfluorooctane sulfonate (PFOS), were determined using a micro-HPLC–MS/MS system, after being subjected to dispersive solid phase extraction (*d*-SPE). The analyzed honey samples originated from seven EU countries (eastern Europe—Poland and Slovakia; southern Europe—Italy, France and Spain; northern Europe—Scotland and England). Due to the necessity of the better research of the Polish retail market, the honey was also collected from industrialized regions of Poland (Malopolska) as well as non-industrial regions (Warmia and Mazury) called “the green lungs of Poland”. Sixteen types of honey collected for the study were as follows: heather, clover, wildflower, multiflorous, linden, rape, buckwheat, forest, honeydew, lemon and orange blossom, thyme, eucalyptus, chestnut, acacia and lavender. These honey samples were representative of three geographic regions of Europe, each differing in climatic, environmental and socio-economic conditions.

## Materials and Methods

According to Commission Recommendation 2010/161/EU, it is required to use a method of analysis that has been proven to generate reliable results. Currently, due to its high sensitivity and selectivity, liquid chromatography hyphenated with tandem mass spectrometry (LC–MS/MS) operating in the multiple reaction monitoring mode (MRM) is the preferred technique for quantitation of PFAS traces (EU Recommendation No. 161 2010). The micro-HPLC technique provides higher peak capacity, greater resolution, increased sensitivity, and a higher speed of analysis compared to the conventional LC system (Guillarme et al. 2010), mainly in combination with MS/MS. Moreover, dispersive solid phase extraction (*d*-SPE) is recommended, involving cleanup using combinations of anhydrous salt and various sorbents to remove interferences. This treatment has been used extensively in the last few years due to its simplicity, speed and effectiveness in cleaning up complex samples (Anastassiades et al. 2003; Surma et al. 2014a, b).

For this work, MS grade reagents, including methanol (MeOH), acetonitrile (MeCN) and formic acid (FA), were purchased from Sigma Chemical Co. (St. Louis, MO, USA). Water was purified with a Milli-Q system (Millipore, Bedford, MA, USA). HPLC grade acetonitrile (for extraction) was purchased from Merck KGaA (Darmstadt, DE). Sodium chloride p.a. and magnesium sulphate anhydrous p.a. were purchased from POCh SA (Gliwice, PL). ENV (styrene–divinylbenzene) SPE bulk sorbent was obtained from Agilent Technologies (Santa Clara, CA, USA). Native (PFBA, PFPeA, PFHxA, PFHpA, PFOA, PFNA, PFDA, PFBS, PFHxS, PFOS) and labelled (L-PFBA, L-PFHxA, L-PFOA, L-PFNA, L-PFDA, L-PFHxS, L-PFOS) PFAS solutions/mixtures were obtained from Wellington Laboratories (Guelph, ON, CA). Stock standard solutions (100 ng mL^−1^) of native and labelled PFASs (IS—internal standard) were prepared in acetonitrile. Working standard solutions (1 ng mL^−1^) of native labelled PFASs were prepared in 20 % MeOH (v/v) with 1 % (v/v) formic acid.

The micro-HPLC system (LC200, Eksigent, Vaughan, ON, CA) consisted of a multi-channel pump, an autosampler (set at 4°C), and a column oven. A system controller coupled with a mass spectrometer (QTRAP 5500, AB SCIEX, Concord, ON, CA) consisting of a triple quadrupole, ion trap and ion source for electro-spray ionization (ESI), and controlled by the Analyst 1.5.1 software, was used to perform the LC–MS/MS analyses. All chromatographic determinations were performed on a HALO C_18_ (50 mm × 0.5 mm × 2.7 µm) column (Eksigent) at 45°C, at a flow rate of 20 μL min^−1^. The compounds were eluted in a gradient system composed of water/formic acid (99.0/1.0, phase A) and acetonitrile/formic acid (99.0/1.0, phase B). The following gradient was used: 40 % B (0–0.5 min), 40 %–90 % B (0.5–3.0 min), 90 % B (3.0–4.0 min), 90 %–40 % (4.0–4.2 min) and 40 % (4.2–5.0 min). Qualitative and quantitative analyses were performed using the multiple reaction monitoring (MRM) method. Optimal identification of analyzed compounds was achieved under the following conditions: negative ionization, curtain gas: 25 L min^−1^, collision gas: 9 L min^−1^, ion spray voltage: −4500 V, temperature: 350°C, 1 ion source gas: 30 L/min, 2 ion source gas: 35 L/min, declustering potential: −30:−85 V, entrance potential: −10 V, collision energy: −10:−65 eV, collision cell exit potential: −10:−38 V. An MPW 351R Centrifuge (MPW Med. Instruments, Warsaw, PL) was used for sample preparation. The vacuum concentrator plus (Eppendorf AG, Hamburg, DE) was used for concentrating extracts.

The honey sample preparation for PFAS-determination, based on *d*-SPE followed by micro-HPLC–MS/MS, was conducted according to the methodology evaluated and validated in a previous study (Surma et al. 2015). Briefly, 5 g of honey was weighed into a 50-mL centrifuge tube, spiked with 250 µl of 100 ng mL^−1^ IS solution (L-PFBA, L-PFHxA, L-PFOA, L-PFNA, L-PFDA, L-PFHxS, L-PFOS), and 5 mL of warm water (50°C) was added to the sample. After cooling to room temperature, 10 mL of acetonitrile (MeCN) and 150 µL of formic acid (FA) were added. The whole tube was vigorously shaken for 1 min, after which 1 g of NaCl and 4 g of MgSO_4_ were added. This was followed by shaking for 1 min, and the solution was finally centrifuged for 15 min at 8700 RCF. Exactly 6 mL of the supernatant was placed in a 15-mL tube, previously prepared with 0.15 g ENV SPE bulk sorbent and 0.900 g MgSO_4_. After 30 s of shaking and 5 min of centrifugation at 5000 RCF, 4 mL of supernatant was transferred into a screw cap vial and evaporated to dryness under a stream of N_2_ at 40°C. Residues were dissolved in 1 mL of methanol. Just before injection, the samples were diluted fivefold in acidified dH_2_O (distillate water with 1 % (v/v) formic acid). Finally, all cleaned-up samples were analyzed by micro-HPLC–MS/MS. Blank samples (to determine recoveries) and reagent blanks were prepared according to the same procedure. Each sample was prepared in triplicate.

A series of standard solutions was prepared in triplicate by diluting the standard mixture solution in 20 % MeOH (v/v) with 1 % (v/v) formic acid in the range of 1–20 ng mL^−1^. Then, 20 µL of the labelled PFAS solution (100 ng mL^−1^) was added to each standard solution.

Food matrices with certified concentrations of perfluoroalkyl substances for honey and related food are not commonly available at the present time. Thus, the usefulness of the method was verified on the basis of the recovery ratio of analyzed compounds (analysis of spiked samples). Recovery studies involved spiking homogenized samples of flower honey with the standard solution of investigated PFASs to a fortification level of 0.001 mg kg^−1^.

The PFASs were identified by retention time (R_t_) and MRM (multiple reaction monitoring) ion pairs. Calibration curves were constructed by calculating the ratio of the peak area versus the peak area of appropriate labelled PFASs against analyte concentration. Investigated analytes were evaluated with the help of assigned labelled PFASs as follows: PFOA/L-PFOA, PFHpA/L-PFOA, PFNA/L-PFNA, PFDA/L-PFDA, PFHxS/L-PFHxS, and PFOS/L-PFOS.

The analyzed samples of honey were purchased from the local market and were originally packed in tightly closed glass jars. Typically collected honey jars ranged in size from 100 to 250 g. The honey samples were stored in a dry place at room temperature. Just after opening, the jar contents were thoroughly mixed until homogeneous, and then 5 g of sample was weighed into a 50-mL centrifuge tube.

Several precautions were taken to avoid cross-contamination from sampling and during analysis. During the entire analytical process, the plastic materials (Eppendorf, centrifuge tube) were sourced only from the Sarstedt Company (Nümbrecht, DE) because these did not show a background signal in subsequent analyses. All plastics and dishes used were disposable, but sterilization was avoided due to the possibility of releasing certain constituents of the plastic materials into stored solutions, which might result in contamination of the sample and an increase in the analytical background. All used dishes were always protected against dust, which can be a source of contamination by perfluoroalkyl substances.

## Results and Discussion

The recovery values, limits of detection (LODs), and limits of quantification (LOQs) determined for PFASs are shown in Table 1. Recovery values of selected PFASs were determined for floral honey samples fortified at a level of 0.001 mg kg^−1^. They ranged from 75 % for PFBA to 93 % for PFNA for all tested analytes.

**Table 1:** The recovery values, LODs, and LOQs for determined PFASs

PFASs Name	Acronym	LOD (ng g^−1^ ww)	LOQ (ng g^−1^ ww)	Recovery (%)
Perfluorobutanoic acid	PFBA	0.023	0.069	75
Perfluoropentanoic acid	PFPeA	0.025	0.075	82
Perfluorobutane sulfonate	PFBS	0.021	0.063	91
Perfluorohexanoic acid	PFHxA	0.015	0.045	87
Perfluoroheptanoic acid	PFHpA	0.017	0.051	91
Perfluorooctanoic acid	PFOA	0.016	0.052	82
Perfluorohexane sulfonate	PFHxS	0.014	0.042	79
Perfluorononanoic acid	PFNA	0.019	0.057	93
Perfluorodecanoic acid	PFDA	0.018	0.054	89
Perfluorooctane sulfonate	PFOS	0.040	0.134	84

The recovery values found for all tested analytes were in good agreement with Commission Recommendation 2010/161/EU, indicating that Member States should carry out the analysis of perfluoroalkylated substances in accordance with Annex III to Regulation (EC) No. 882/2004 of the European Parliament and of the Council of 29 April 2004 on official controls performed, to ensure verification of compliance with feed and food laws and animal health and welfare rules, by making use of a method of analysis that has been proven to generate reliable results. Ideally, the recommended recovery rates should be within the range of 70 %–120 %.

In this study, LOQ values for all tested perfluoroalkyl substances ranged from 0.042 ng g^−1^ ww for PFHxS to 0.134 ng g^−1^ ww for PFOS. The obtained values are in good agreement with the LOQ value (1 µg kg^−1^) recommended by Commission Recommendation 2010/161/EU.

A breakdown of the concentrations of the studied compounds found in the studied samples is shown in Table 2. The results are the mean and standard deviations of three independent extractions (n = 3).

**Table 2:** Content of selected PFASs in analyzed honey samples (ng g^−1^ ww)

Honey sample		PFCAs								PFSAs			
		PFOA		PFHpA		PFNA		PFDA		PFHxS		PFOS	
Type	Country of origin	Mean	SD	Mean	SD	Mean	SD	Mean	SD	Mean	SD	Mean	SD
Heather	Scotland	0.221	0.017	nd	–	nd	–	nd	–	nd	–	nd	–
	England A	0.295	0.023	0.413	0.008	nd	–	nd	–	nd	–	nd	–
	England B	nd	–	0.287	0.006	nd	–	nd	–	nd	–	nd	–
	Spain	0.223	0.014	nd	–	0.253	0.021	nd	–	0.191	0.014	<LOQ	–
Clover	Scotland	nd	–	0.317	0.023	nd	–	nd	–	nd	–	nd	–
Wildflower	England A	0.124	0.007	nd	–	nd	–	nd	–	nd	–	nd	–
	England B	nd	–	nd	–	nd	–	nd	–	nd	–	nd	–
Multiflorous	Poland M	nd	–	0.218	0.001	nd	–	nd	–	nd	–	nd	–
	Poland W	nd	–	0.183	0.002	nd	–	nd	–	nd	–	nd	–
	Scotland	0.134	0.013	–	–	nd	–	nd	–	nd	–	nd	–
	Slovakia	nd	–	0.170	0.015	nd	–	nd	–	nd	–	nd	–
Linden	Poland M	nd	–	0.353	0.028	nd	–	nd	–	nd	–	nd	–
	Poland W	nd	–	0.263	0.012	nd	–	nd	–	nd	–	nd	–
	France	nd	–	nd	–	nd	–	nd	–	nd	–	nd	–
Rape	Poland M	nd	–	0.443	0.026	nd	–	nd	–	nd	–	nd	–
	Slovakia	nd	–	0.201	0.007	nd	–	nd	–	nd	–	nd	–
Buckwheat	Poland W	nd	–	0.395	0.010	nd	–	nd	–	nd	–	nd	–
Forest	Slovakia	nd	–	0.203	0.005	nd	–	nd	–	nd	–	nd	–
Honeydew	Slovakia	nd	–	0.190	0.005	nd	–	nd	–	nd	–	nd	–
Lemon blossom	Spain	0.326	0.010	0.135	0.012	0.174	0.017	nd	–	0.116	0.008	nd	–
Orange blossom	Spain	0.047	0.004	nd	–	nd	–	nd	–	nd	–	nd	–
Thyme	Spain	0.167	0.004	0.309	0.010	0.071	0.004	nd	–	0.132	0.012	nd	–
Eucalyptus	Italy	0.121	0.002	0.250	0.001	0.149	0.010	0.278	0.019	0.080	0.002	<LOQ	–
Chestnut	France	0.345	0.012	nd	–	nd	–	nd	–	<LOQ	–	nd	–
Acacia	France	0.103	0.005	nd	–	0.113	0.003	nd	–	nd	–	nd	–
Lavender	Spain	0.162	0.014	nd	–	0.225	0.021	nd	–	nd	–	<LOQ	–

PFBA, PFPeA, PFHxA and PFBS were not detected in any honey samples

*SD* standard deviation, *nd* not detected A, *B* brand of honey, *M* honey from Malopolska region, *W* honey from Warmia and Mazury region

**Fig. 1:**
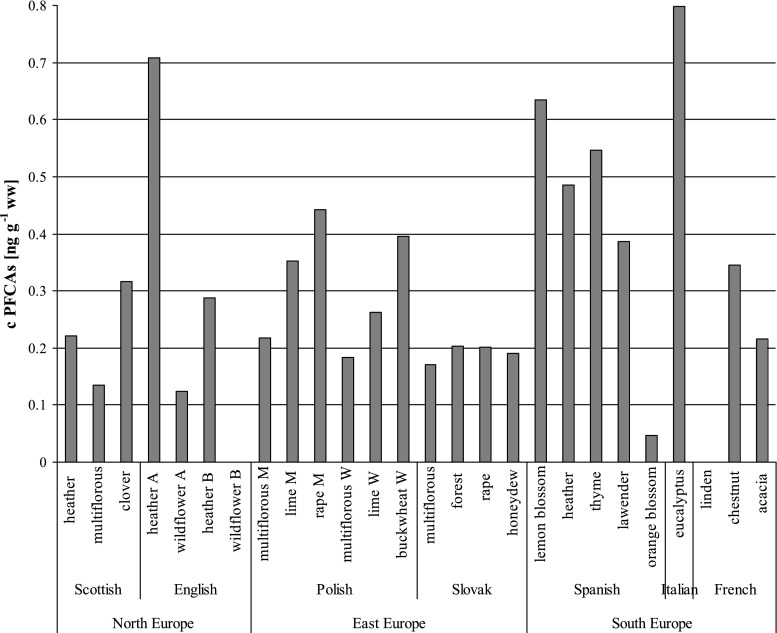
The total content of PFCAs in investigated honey

Apart from three honey samples (English wildflower B, Spanish orange blossom and French linden), perfluoroalkyl carboxylic acids (PFCAs) were detected in almost all analyzed honey samples in the range from 0.124 ng g^−1^ ww for English wildflower A to 0.798 ng g^−1^ ww for Italian eucalyptus, which is presented in Fig. 1.

The percent values of samples (%) in which particular PFCAs were found increased in the following order: PFDA (3.8) < PFNA (23.1) < PFOA (46.2) < PFHpA (61.5). PFHpA was mostly detected in Polish and Slovak honey samples with a mean of 0.309 and 0.191 ng g^−1^ ww, respectively. It was also found in individual samples of Scottish (clover), English (heather), Spanish (lemon blossom and thyme), and Italian (eucalyptus) honey. PFOA was primarily quantified in Spanish honey samples with a mean of 0.187 ng g^−1^ ww. It was also present in samples from Scotland (heather and multiflorous), England (heather and wildflower), Frence (chestnut and acacia), and Italy (eucalyptus). PFNA as well as PFOA were primarily determined in honey from Spain, with values in the range from 0.071 ng g^−1^ ww for thyme to 0.253 ng g^−1^ ww for heather. Moreover, it was identified in French acacia (0.113 ng g^−1^ ww) and Italian eucalyptus (0.149 ng g^−1^ ww) honey. PFDA was quantified in only one honey sample, Italian eucalyptus, at a level of 0.278 ng g^−1^ ww. Among perfluoroalkane sulfonates (PFSAs), only PFHxS was quantified in four honey samples and ranged from 0.080 ng g^−1^ ww to 0.191 ng g^−1^ ww for Italian eucalyptus and Spanish heather, respectively. PFOS was detected in only three honey samples (Italian eucalyptus, and Spanish heather and lavender); however, the concentrations were below LOQ. Italian eucalyptus honey was characterized by the highest variety and total content of PFASs.

Considering the honey type, the level of contamination with PFOA in heather honey was the same in samples from Scotland, England, and Spain. PFHpA was detected only in English honey, with a higher amount in the A brand sample. PFNA (0.253 ng g^−1^ ww) and PFHxS (0.014 ng g^−1^ ww) were only identified in Spanish honey. Only two PFASs were found in multiflorous honey: PFOA in the Scottish sample (0.134 ng g^−1^ ww) and PFFpA in the Slovak sample (0.170 ng g^−1^ ww) and in both Polish honey samples, from Malopolska (0.218 ng g^−1^ ww) and the Warmia and Mazury (0.183 ng g^−1^ ww) regions. In linden honey samples, also originating from these two Polish regions, the PFHpA concentration was 0.353 and 0.263 ng g^−1^ ww, respectively. The results indicate that honey samples from the two Polish regions are diverse in terms of contaminated by PFASs. A higher concentration of PFCAs was noticed in Malopolska (industrialized region of Poland) honey compared to that from Warmia and Mazury (regarded as the least polluted region of Poland, also called the “green lungs of Poland”).

From the perspective of regions of origin for the various honey samples (eastern Europe—Poland and Slovakia; southern Europe—Italy, France and Spain; northern Europe—Scotland and England), it was observed that the level of PFCA contamination slightly increased in the following order: northern European countries < eastern European countries < southern European countries. However, examining the average content of perfluoroalkyl carboxylic acid in these parts of Europe, no statistically significant differences were observed. Values of 0.256 ± 0.227 ng g^−1^ ww, 0.262 ± 0.099 ng g^−1^ ww and 0.385 ± 0.265 ng g^−1^ ww were obtained for northern, eastern and southern European countries, respectively. In contrast to the results obtained from the two regions of Poland, the honey samples originating from three geographic regions of Europe, differing in climatic, environmental, and socio-economic conditions, showed no regional impact on PFAS contamination levels.

A review of the literature revealed very little information about the contamination of honey with PFASs. PFOA and PFOS in four Italian honey samples from the Mount Amiata area were analyzed by Guerranti et al. (2013). No positive results were obtained. The investigated analytes were below the detection limit (0.5 ng g^−1^ ww). According to the Scientific Report of EFSA (EFSA 2011) on the results of monitoring perfluoroalkylated substances in food during the period 2000-2009, 30 honey samples were tested for PFOA and PFOS content. The level of PFOA ranged between 0.25 and 0.47 ng g^−1^. Another EFSA Scientific Report (EFSA 2012) covered the occurrence and dietary exposure to perfluoroalkylated substances via food. The above assessment was based on 54195 analytical results obtained for 7560 food samples covering a list of 27 PFASs, but not all samples were analyzed for the full set of PFASs. The data were submitted by 13 European countries for samples collected in the period 2006-2012. Four of 39 honey samples analyzed for PFOA provided quantifiable results in the range from 2 to 470 ng kg^−1^. PFDA was quantified in one honey sample (8 ng kg^−1^). Similarly, PFHxA (24 ng kg^−1^) and PFOS (55 μg kg ^−1^) were found only in one sample.

In our study, the quantity of PFOAs was found to be in the range from 0.047 ng g^−1^ for Spanish orange blossom to 0.345 ng g ^−1^ for French chestnut. We can conclude that PFOA content was at a similar level in all studies. PFDA was also quantified only in one honey sample of Italian eucalyptus (0.278 ng g ^−1^), but its content was approximately 35 times higher than that reported by the EFSA (EFSA, 2012). PFHxA and PFOS were not quantified in this study.

An analytical method based on dispersive solid phase extraction (*d*-SPE) and micro-HPLC–MS/MS detection was successfully applied for determination of two main groups of PFASs in honey samples: perfluoroalkyl carboxylic acids (PFCAs) and perfluoroalkane sulfonates (PFSAs). The efficient use of the modified QuEChERS method with a polymer-based sorbent—ENV for efficient honey sample preparation—was demonstrated. The presented method is suitable for determination of PFASs in honey and can be extended to other food samples. It can be concluded that the presence of perfluoroalkyl substances in honey may serve as an indicator of environmental pollution.
